# Biodiversity Assessment Using Hierarchical Agglomerative Clustering and Spectral Unmixing over Hyperspectral Images

**DOI:** 10.3390/s131013949

**Published:** 2013-10-15

**Authors:** Ollantay Medina, Vidya Manian, J. Danilo Chinea

**Affiliations:** 1 Computing and Information Sciences and Engineering, University of Puerto Rico at Mayaguez, Call box 9000, Mayaguez 00681, Puerto Rico; 2 Department of Electrical & Computer Engineering, University of Puerto Rico at Mayaguez, Call box 9000, Mayaguez 00681, Puerto Rico; E-Mail: manian@ece.uprm.edu; 3 Department of Biology, University of Puerto Rico at Mayaguez, Call box 9000, Mayaguez 00681, Puerto Rico; E-Mail: jdchinea@yahoo.com

**Keywords:** hyperspectral images, biodiversity, hierarchical clustering

## Abstract

Hyperspectral images represent an important source of information to assess ecosystem biodiversity. In particular, plant species richness is a primary indicator of biodiversity. This paper uses spectral variance to predict vegetation richness, known as Spectral Variation Hypothesis. Hierarchical agglomerative clustering is our primary tool to retrieve clusters whose Shannon entropy should reflect species richness on a given zone. However, in a high spectral mixing scenario, an additional unmixing step, just before entropy computation, is required; cluster centroids are enough for the unmixing process. Entropies computed using the proposed method correlate well with the ones calculated directly from synthetic and field data.

## 1. Introduction

Hyperspectral images represent an important tool to assess ecosystem biodiversity. Improvements in spectral and spatial sensors resolution allow more precise analysis of biodiversity indicators that should agree with indicators obtained using field data. The development of accurate analysis tools would be advantageous to extend the analysis to larger zones by hyperspectral image processing; furthermore, for future scenarios, given that the actual data gathering is expensive in time and resources, a pre-analysis would give extra knowledge for planning a more effective campaign to collect field data.

One of the most important indicators of biodiversity in ecosystems is plant species richness. This indicator can be measured in hyperspectral images considering the Spectral Variation Hypothesis (SVH) proposed in [1], SVH states that spectral heterogeneity is related to spatial heterogeneity and thus to species richness. Subsequent efforts to validate SVH can be found in [2,3]. This paper aims to capture spectral heterogeneity by means of hierarchical agglomerative clustering and then use the result for prediction of plant species richness. The information of heterogeneity can be used to compute entropies like Shannon, Gini-Simpson or Renyi among others [4]; in this study, Shannon entropy is chosen, given that it is commonly used in biodiversity. There are other studies that try to capture plant diversity starting from spectral heterogeneity as in [5,6], these studies estimate the spectral variability using the radius of hyperspectral clusters, this variability is related to the alpha diversity [4], a measure of the biological diversity of a particular community, habitat or sampling unit.

The main contribution of this paper is the focus on proportional abundances that entropy indexes can capture which are more meaningful than species richness alone. It also evaluates the use of hierarchical clustering to assess biodiversity and the use of spectral unmixing to improve results.

This paper is organized as follows: Section 2 gives a brief theoretical background. Section 3 explains the methodology used to process hyperspectral imagery. Section 4 explains the details of experiments and gives the results. Section 5 discusses the experimental results. Section 6 presents the conclusions.

## 2. Theoretical Background

### 2.1. Hierarchical Clustering

The goal of Hierarchical Clustering is to build a hierarchy of clusters, a cluster tree. There are two main approaches to build a hierarchy of clusters: Agglomerative and divisive. The former approach is used in this work. Hierarchical Agglomerative Clustering (HAC) is a “bottom up” approach in which, at first, each observation constitutes its own cluster; then, pairs of clusters are merged successively, generating the hierarchy, until there is just one cluster left. HAC needs two concepts to carry out the process: a metric (distance) and a linkage [7]. The metric measures the dissimilarity between pairs of observations. The linkage measures the dissimilarity between clusters as a function of the pairwise distances of observations in compared clusters. HAC can work directly with the dissimilarity matrix, a representation just in terms of dissimilarity between pairs of objects. The dissimilarity matrix is an *n*x*n* matrix *D*, where *n* is the number of observations. An entry d_ij_ in *D* represents the dissimilarity between the *i*th and *j*th observations. In subsequent sections the notation used considers *x* or *y* as individual observations, more specifically, pixels; and *X* or *Y* as groups (clusters) of pixels.

### 2.2. Metrics

Metrics used to construct the dissimilarity matrix:

Euclidean distance:
  (1) d(x,y)=‖y−x‖
Spectral angle distance (SAD):
  (2) $$d(x,y)=\cos^{-1}(\frac{x.y}{\left\|x\|\;\|y\right\|})$$

where x and y are pixels, ‖·‖ is the Euclidean norm and the dot operation is the usual dot product between vectors.

### 2.3. Linkage

The linkage used in this work is known as complete-linkage [7]. This linkage returns compact clusters of similar size:

(3) $$d(X,Y)=\max_{x\in X,y\in Y}d(x,y)$$

where X and Y are clusters.

### 2.4. Number of Clusters

**Figure 1.**
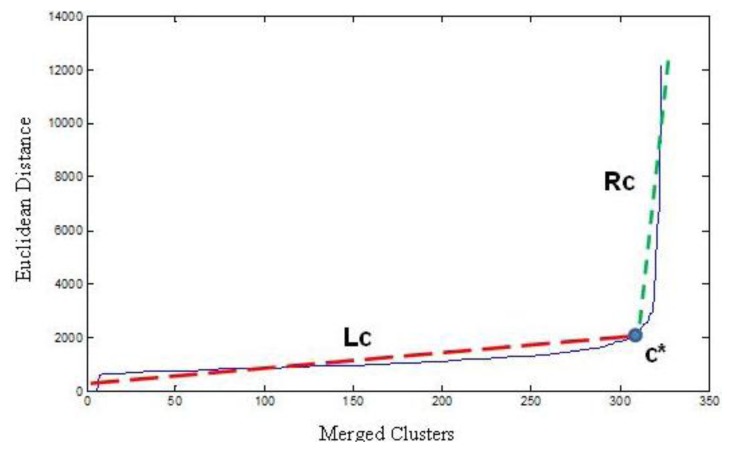
Determining number of clusters using critical point “c*”.

Every time HAC has to merge two clusters, it chooses clusters that have minimal distance. Merging n observations requires n−1 operations. This n−1 linkage valued sequence produce a characteristic “elbow” curve. Obtaining a number of clusters in a non-supervised fashion requires choosing a point in the “elbow” part of the curve. The Number of Clusters (NC) is determined using the method in [8], a critical point c* is chosen such that:

(4) $$c*=\arg\min_{c}({\text{RMSE}}_{c})$$

(5) $${\text{RMSE}}_{c}=\frac{c}{n}\text{RMSE}(L_{c})+\frac{n-c}{n}\text{RMSE}(R_{c})$$

where RMSE is the root mean squared error, and L_c_ and R_c_ are best-fit lines. Figure 1 shows an “elbow” curve, the L_c_ line covers intra-cluster linkages; the R_c_ line covers inter- cluster linkages.

### 2.5. Shannon Entropy

The Shannon entropy is a popular biodiversity index that represents the weighted geometric mean of the proportional abundances of the species [4]. It is calculated as follows:

(6) $$h=-\sum_{i=1}^{k}p_{i}\ln p_{i}$$

where k is the number of species and p_i_ is the proportion of abundance of the ith species. Applying Shannon entropy to spectral data requires grouping pixels under some criteria. Otherwise, it is very likely that every pixel is unique, rendering every p_i_ to 1/k; which would produce a constant and also meaningless maximum entropy *h* = ln(*k*).

### 2.6. Pearson's Correlation Coefficient

Pearson's correlation coefficient between two variables is defined as the covariance of the two variables divided by the product of their standard deviations [9]:

(7) $$\rho_{U,V}=\frac{\mathrm{cov}(U,V)}{\sigma_{U}\sigma_{V}}$$

### 2.7. Spectral Unmixing

In a hyperspectral image, the reflected or emitted radiation measured per pixel, rarely comes from the interaction with a single homogenous material. If the spatial resolution is higher than the size of an object in the image, it is very likely that the image have pure pixels, though this case is not frequent.

There are two models to analyze the mixing problem, linear model and non-linear model. For simplicity, only the linear mixing model [10] is covered briefly. If spectral signatures of all pure materials in the image are known, then the linear mixing model assumes that every pixel in the image is the result of a linear combination of spectral signatures of each material, and some noise:

(8) $$I_{\left(b\times n\right)}=S_{\left(b\times p\right)}A_{\left(p\times n\right)}+M_{\left(b\times n\right)}$$

where *I* is the pixels matrix, *S* is the endmembers matrix, *A* is the abundances matrix and *M* is the noise matrix. There are n pixels, p endmembers and b bands. Abundances are normalized such that, for a given pixel j in *I*:

(9) $$\sum_{i=1}^{p}a_{ij}\leq 1$$

Frequently, there are spectral signature differences, even among pure pixels of the same material, attributable to shadows or different reflection angles that weaken measurements; the linear mixing model accounts these cases with abundances in Equation (9) strictly less than one.

The Spectral Unmixing (SU) approach used in this study computes matrix *S* first, using a maximum distance (MaxD) algorithm [11]; then, computes matrix *A* using a non-negative least-squares (NNLS) algorithm [10]. For this approach, the number of endmembers is required. In this work, the number of endmembers is obtained from field data.

Since the proposed method is aimed at capturing the Shannon entropy for a set of pixels. The ideal case occurs when all pixels are pure ones; in this case, each cluster represents specific species clearly. The typical case is when a pixel represents not one, but a combination of different species; in this case, clusters are less useful to capture species diversity since several species can be grouped in the same cluster. The unmixing process decomposes the spectral combination of species into abundances for every pixel; these abundances can be used as in the ideal case to compute entropy.

In the following sections, terms like high, mid or low spectral mixing are used. These terms refer to the proportion in spectral data between pixels that are a combination of endmembers *versus* pixels that are almost pure ones. In a high spectral mixing scenario, most pixels possess spectral mixing; in a low spectral mixing scenario, most pixels are almost pure.

## 3. Methodology

### 3.1. Image Preprocessing

This stage prepares the hyperspectral image by removing duplicate pixels, and non-vegetation pixels. Duplicate pixels can result from the nearest-neighbor resampling of image pixels during some geo correcting procedures. Duplicates removal is efficiently achieved using a radix-sort based algorithm [12], where every pixel is treated as a number and bands are treated as digits; sorted pixels are scanned sequentially to remove, or mark, duplicates. Non-vegetation pixel removal is achieved through NDVI index computation and choosing a threshold to discard pixels with values below the threshold.

### 3.2. Image Partition

The hyperspectral image is partitioned in a set of rectangular parts. It is not mandatory that all rectangles have the same dimensions. However, it is advisable that the number of pixels contained in rectangular elements is as uniform as possible; this ensures that possible entropy values are in the same range, making comparisons between entropy values, in different rectangular elements, more meaningful.

### 3.3. Algorithm

This algorithm constitutes the core procedure of the proposed method. The algorithm deals successfully with a general spectral mixing scenario, under the requirement that the number of endmembers is provided.

Input: *L*, an image partition, read as a list of sets of hyperspectral pixels. *M*, a list containing number of endmembers for elements in *L*.

Output: *H*, a list of Shannon entropy values corresponding to elements in *L*.

For each partition element *l* in *L*:

Step 1. Apply HAC to *l* to produce a cluster tree *T*.
Step 2. Apply NC to *T* to establish critical point c*.
Step 3. From *T*, and using c*, extract group of clusters *G*.
Step 4. From *G*, obtain group of centroids *G'*; every cluster *g* in *G* has a corresponding centroid *g'* in *G'*. Using corresponding number of endmembers *m* in *M*, apply SU to *G'* to produce abundances matrix *A*.
Step 5. Compute Shannon entropy *h* of *l*; add *h* to *H. h* is computed using Equation (6) making *k* = *m. p_i_* represents the probability of the *i*th endmember, computed using Equation (10):
  (10) $$p_{i}=\frac{1}{n}\sum_{j=1}^{\left|G\right|}a_{ij}\left|g_{j}\right|$$
  where *n* is the number of pixels, |*G*| is the number of clusters, *a_ij_* is the *i*th endmember abundance in *j*th cluster centroid, |*g_j_*| is the number of pixels in the *j*th cluster. All of these values are taken from the current *l* in *L*.

There is a variant to this algorithm that could be used in particular cases, when data exhibits low or mid spectral mixing. The benefit of the variant is that spectral unmixing is completely avoided; resorting only to cluster information to compute entropy and doing so in a truly automatic fashion. Unfortunately, deciding when to use this variant is very circumstantial requiring expert human judgment to apply it. Following is the variant, which reuses most of the notation of the previous algorithm.

Input: *L*.

Output: *H*.

For each partition element *l* in *L*:

Step 1. Apply HAC to *l* to produce a cluster tree *T*.
Step 2. Apply NC to *T* to establish critical point c*.
Step 3. From *T*, and using c*, extract group of clusters *G*.
Step 4. Compute Shannon entropy *h* of *l*; add *h* to *H. h* is computed using Equation (6) making *k* = |*G*|. *p_i_* represents the probability of the *i*th cluster, computed using Equation (11),
  (11) $$p_{i}=\frac{\left|g_{i}\right|}{n}$$
  where |*g_i_*| is the number of pixels in the *i*th cluster, *n* is the number of pixels. All of these values are taken from the current *l* in *L*.

### 3.4. Image Reconstruction

**Figure 2.**
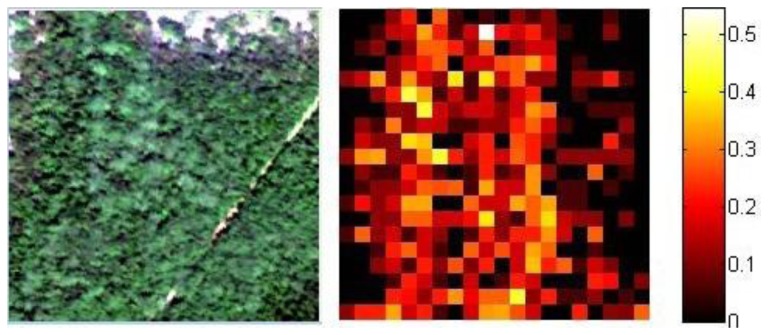
(**Left**) A portion of the Guanica forest, size 200 × 200 pixels; (**Right**) Computed Shannon entropies using the proposed method, zone size 10 × 10 pixels. Higher entropy means more proportional spectral heterogeneity.

Every part in the original partition is replaced with its respective Shannon entropy index; the value is replicated to match the original size of the partition element, then the new image is reassembled as shown in Figure 2.

## 4. Experimental Results

The experiments tried to verify that the proposed method computes a biodiversity index, which reflects to a certain degree the species richness in the hyperspectral image. Two kinds of experiments are conducted; the first experiment uses synthetic hyperspectral data to assess the influence of spectral mixing, to verify proper operation and to identify limitations of the proposed method. The spectral mixing degree refers to the portion of non-pure pixels in the dataset. The second experiment uses real hyperspectral data along with field data to assess the index of correlation between the two datasets.

### 4.1. Synthetic Hyperspectral Data

Data is generated assuming a linear mixing model. Endmembers spectra come from an independent unmixing process. All endmembers represent vegetation. Considering the notation introduced in Section 2.7, Equations (12) and (13) depict the specifics of the model used:

(12) $$I_{\left(b\times n\right)}=S_{\left(b\times p\right)}A_{\left(p\times n\right)}$$

(13) $$0.9\leq \overset{p}{\underset{i=1}{\text{∑}}}a_{ij}\leq 1$$

where *n* = 1000 pixels, *p* = 5 endmembers chosen randomly from a set of 10 endmembers on every trial, *b* = 989 bands. Abundances matrix *A* is randomly generated on every trial; generation of *A* obeys three criteria: Equation (13), a fraction *r* of pixels with spectral mixing, and using at most 3 endmembers for spectral mixing. *r* is the truly changing parameter along the trials that substantially affects the results. Changes in *p* or the number of endmembers to mix didn't have much influence.

Real entropy index of synthetic pixels is computed using matrix *A*. Matrix *I* is passed as input to the algorithm in Section 3.3, which returns the proposed entropy index. After 20 trials, for every set of parameters, correlation between results is computed. Results consider an only-clustering case and clustering-unmixing case. Table 1 shows these results.

### 4.2. Real Hyperspectral Data and Field Data

The data is from the Guanica dry forest in Puerto Rico, the field data is from 25 circular plots, each plot has a radius of 10 m. The sampling was conducted between 2006 and 2009; sample locations were chosen at random and stratified by history of land use and topographic position. Field data considered vegetation, specifically plants with stem radius larger than 5 cm. The corresponding hyperspectral images are from AISA airborne imagery acquired in 2007, the images have 128 bands, wavelengths from 400–1000 nm, and spatial resolution of 1 m. The data is divided in four groups, because there are four separate hyperspectral images taken at different times producing differences in reflectance.

**Figure 3.**
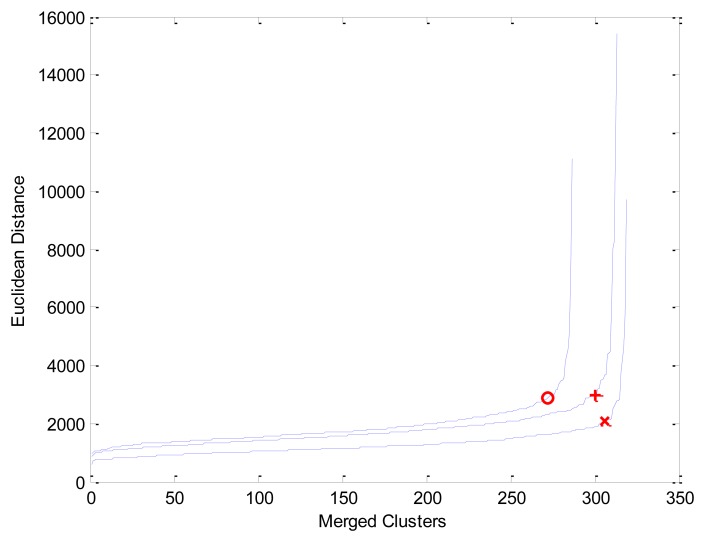
Three instances of “elbow” curves produced during HAC process using real hyperspectral data. Corresponding critical points c* in: “o” = (272, 2883) produced 16 clusters, “+” = (300, 2970) produced 14 clusters and “×” = (306, 2079) produced 14 clusters.

Under these specifications, every hyperspectral zone had 324 pixels, where every pixel is a vector of 128 components. Figure 3 shows three instances of “elbow” curves and corresponding critical points c* (markers “o”, “+” and “×”) used by HAC to compute clusters; after duplicate and non-vegetation pixels discarding, curves show: marker “o” coordinates (272, 2883) produced 16 clusters in a zone of 288 pixels, marker “+” coordinates (300, 2970) produced 14 clusters in a zone of 314 pixels and marker “×” coordinates (306, 2079) produced 14 clusters in a zone of 320 pixels.

The number of endmembers for the unmixing stage is set up corresponding to the species richness of the plot, also collected with field data. The proposed method processes the whole data batch in 30 s approximately. The validation of the proposed index is done calculating the Pearson correlation coefficient between the Shannon entropy of actual field data and the Shannon entropy computed in the corresponding image using the algorithm in Section 3.3. Table 2 shows the results.

## 5. Discussion

**Figure 4.**
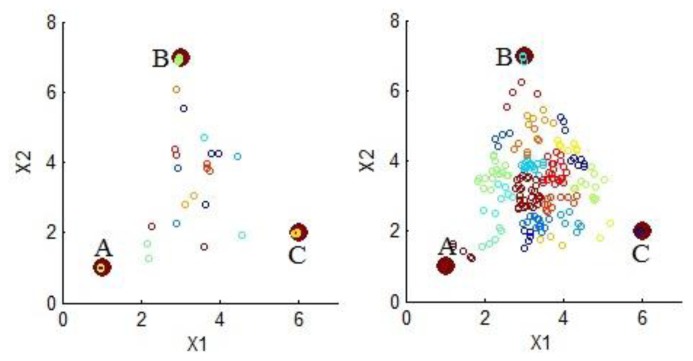
(**Left**) A set of points with low spectral mixing, most of the points are close to the endmembers; (**Right**) A set of points with high spectral mixing, most of the points are not close to the endmembers. In both cases, colors represent how the clustering process group the points.

The effect of spectral mixing over HAC can be better appreciated in a simplified hypothetical example, using only three endmembers, one hundred points, two bands and two degrees of spectral mixing, as depicted in Figure 4; where clusters are represented using points with same color. The left side of the figure shows a set of points with low spectral mixing; in this case, important clusters are around the endmembers. Thus, most of the entropy is directly captured by these clusters. The right side of the figure shows a set of points with high spectral mixing; in this case, important clusters do not represent specific endmembers but a mix of them. Thus, entropy cannot be captured from these clusters unless an unmixing process is applied.

First experiment confirmed how degree of spectral mixing of data affects the proposed method. HAC gives results that are acceptable or even good with a medium mixing degree or less. The worst case appears in a high spectral mixing scenario, where important clusters are composed by non-pure pixels, and less populated clusters are those related to pure pixels. This limitation cannot be avoided even with more sophisticated hierarchical clustering methods. SAD has advantage over Euclidean distance if HAC alone is used. Using HAC and SU in most cases improve the results; in this case, Euclidean distance has advantage over SAD. The downside of using spectral unmixing is that, at least, the number of endmembers is needed, or even endmembers data themselves; this information is not necessarily known and its computation is not straightforward [13]. As a heuristic, one could expect medium or low spectral mixing if spatial resolution is higher than the size of same-material objects in the hyperspectral image.

Second experiment shows positive correlation between field data entropy and computed entropy almost exclusively using HAC and SU. Given that spectral data comes from vegetation in a forest, high spectral mixing is the most likely scenario; followed by mid spectral mixing in zones having plants with big (greater than spatial resolution) crown surface or dense population. Negative correlations for HAC can be explained as being due to few important clusters containing information of several endmembers, species with very similar spectral signatures or uniform compounds of vegetation. Low correlation in Group 4 can be explained by presence of vegetation with stem radius below the 5 cm. threshold.

## 6. Conclusions

A method based on hierarchical agglomerative clustering and spectral unmixing is presented, this method captures spectral variability of vegetation in hyperspectral images; the captured information could be used to assess plant species richness. The method works well even in scenarios with high spectral mixing, but requires a way to determine the number of endmembers or endmembers themselves. Experimental results show positive correlation between the Shannon entropy of the field data and the one computed using the proposed method.

## Figures and Tables

**Table 1.** Entropy correlation using *p* = 5 endmembers and increasing fraction *r* of data with spectral mixing.

**Spectral Mixing (r)**	**Euclidean**		**SAD**	
	
	**HAC**	**HAC&SU**	**HAC**	**HAC&SU**
0.1	0.64	0.98	0.95	0.99
0.2	0.54	0.99	0.92	0.73
0.3	0.75	0.87	0.83	0.55
0.4	0.43	0.95	0.39	0.40
0.5	0.48	0.98	0.67	0.55
0.6	0.59	0.98	0.58	0.87
0.7	0.43	0.61	0.50	0.56
0.8	0.43	0.99	0.07	0.64
0.9	0.11	0.72	0.31	0.37

**Table 2.** Entropy correlation between field data and proposed method.

**Spectral Data**	**Euclidean**		**SAD**	
	
	**HAC**	**HAC&SU**	**HAC**	**HAC&SU**
Group 1	−0.19	0.85	−0.12	0.51
Group 2	0.59	0.78	0.65	0.87
Group 3	0.50	0.82	−0.06	0.87
Group 4	−0.56	0.30	−0.28	0.41

## References

[1] Palmer M.W., Wohlgemuth T., Earls P., Arévalo J.R., Thompson S.D. Opportunities for Long-Term Ecological Research at the Tallgrass Prairie Preserve, Oklahoma.

[2] Palmer M.W., Earls P.G., Hoagland B.W., White P.S., Wohlgemuth T. (2002). Quantitative tools for perfecting species lists. Environmetrics.

[3] Rocchini D., Chiarucci A., Loiselle S.A. (2004). Testing the spectral variation hypothesis by using satellite multispectral images. Acta Oecol..

[4] Jost L. (2006). Entropy and diversity. Oikos.

[5] Rocchini D. (2007). Effects of spatial and spectral resolution in estimating ecosystem α-diversity by satellite imagery. Remote Sens. Environ..

[6] Rocchini D., Balkenhol N., Carter G.A., Foody G.M., Gillespie T.W., He K.S., Kark S., Levin N., Lucas K., Luoto M. (2010). Remotely sensed spectral heterogeneity as a proxy of species diversity: Recent advances and open challenges. Ecolo. Inform..

[7] Hastie T.J., Tibshirani R.J., Friedman J.H. (2005). The elements of statistical learning: Data mining, inference, and prediction. Math. Intell..

[8] Salvador S., Chan P. Determining the Number of Clusters/Segments in Hierarchical Clustering/Segmentation Algorithms.

[9] Lee Rodgers J., Nicewander W.A. (1988). Thirteen ways to look at the correlation coefficient. Am. Stat..

[10] Keshava N., Mustard J.F. (2002). Spectral unmixing. IEEE Signal Process. Mag..

[11] Parente M., Plaza A. Survey of Geometric and Statistical Unmixing Algorithms for Hyperspectral Images.

[12] Leiserson C.E., Rivest R.L., Stein C., Cormen T.H. (2001). Introduction to Algorithms.

[13] Ambikapathi A., Chan T.H., Chi C.Y., Keizer K. (2013). Hyperspectral data geometry-based estimation of number of endmembers using p-norm-based pure pixel identification algorithm. IEEE Trans. Geosci. Remote Sens..

